# Enhanced Polaron Delocalization in Phytic Acid-Doped Polyaniline Nanofibers

**DOI:** 10.1021/acs.chemmater.6c00240

**Published:** 2026-08-03

**Authors:** Laraib Nisar, Martin V. Appleby, Abeera Hassan, Benedict J. Smith, Dimitri Chekulaev, Qizhi Ba, Mohamed Pourkashanian, Mohammed S. Ismail, Adrien A. P. Chauvet

**Affiliations:** † Dainton Building, Department of Chemistry, The University of Sheffield, Brook Hill, Sheffield, South Yorkshire S3 7HF, United Kingdom; ‡ Paul Scherrer Institut, Forschungsstrasse 111, Villigen PSI 5232 Switzerland; § Department of Chemistry, 6752 Brown University, 324 Brook Street, Providence, Rhode Island 02912, United States; ∥ Energy 2050, Department of Mechanical Engineering, Faculty of Engineering, University of Sheffield, Sheffield S3 7RD, United Kingdom; ⊥ Translational Energy Research Centre, The University of Sheffield, Sheffield S9 1ZA, United Kingdom; # School of Engineering, 4019 University of Hull, Hull HU6 7RX, United Kingdom

## Abstract

Polyaniline is a widely used conducting polymer because of its facile synthesis and unique optical and electrochemical properties. It has been demonstrated that protonation of polyaniline increases its conductivity by inducing the formation of polarons along the polymer backbone. These polarons are responsible for charge transport through the conjugated system. In this work, we investigate the impact of phytic acid (PA) doping on the electronic properties of polyaniline (PANi) synthesized via the oxidative polymerization method. The photophysical properties were studied using ultrafast transient absorption spectroscopy (TAS). Compared to undoped PANi, phytic acid-doped polyaniline (PA/PANi) exhibits enhanced charge carrier delocalization and prolonged polaron lifetimes, which indicates improved charge transport dynamics. The photophysical behavior observed at 300, 400, and 600 nm pump wavelengths shows the significant spectral changes that are attributed to protonation and cross-linking between PA and PANi. This enhanced charge carrier mobility is due to the formation of a three-dimensional (3D) interconnected mesh-like nanofiber structure upon PA doping. These findings provide insights into how molecular-level doping can be used to tune charge transport properties in conducting polymers, with potential applications for the design of advanced organic electronic and energy storage devices.

## Graphical abstract

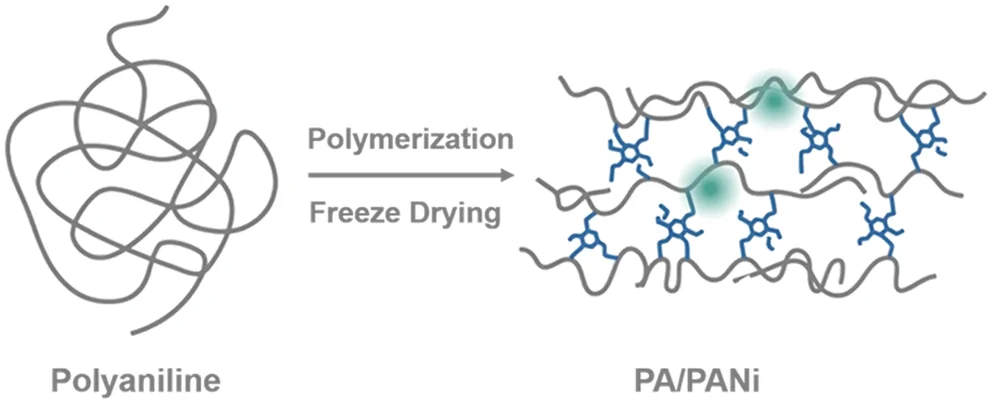

## 1 Introduction

Conducting polymers have gained significant attention since their discovery due to their unique structural and optical properties.^1,2^ Polyaniline (PANi) stands out among the conducting polymers for its low cost and facile synthesis with diverse applications in optoelectronic devices, sensors, and energy storage.^3–5^ PANi exists in three primary oxidation states, the fully reduced leucoemeraldine base, the half-oxidized emeraldine base, and the fully oxidized pernigraniline form. Of these, the emeraldine base is the most stable form under ambient conditions.^6^ However, emeraldine base is poorly conductive in its neutral state. The conductivity of PANi arises from protonation of the emeraldine base with acids, converting it into the emeraldine salt form and generating mobile charge carriers.^7,8^ By varying the protonic acid dopant, these charge carriers can be tuned, allowing control over the electrical conductivity through the formation of polaronic defect states.^9^

Polarons are quasiparticles that form when protonation of the imine nitrogen sites in polyaniline induces charge redistribution along the π-conjugated backbone of PANi, resulting in an unpaired electron and a locally distorted polymer backbone. This local structural relaxation is accompanied by a redistribution of bonds along the chain, giving rise to a mixed benzenoid-quinoid electronic configuration rather than a full transformation between the two forms. Together, the unpaired electron and the associated local geometric distortion constitute the polaron.^10^ Polarons play a crucial role in the electrical and optical properties of doped PANi by acting as mobile charge carriers and facilitating conductivity through hopping or tunnelling between localized states along and between polymer chains.

So far, several different kinds of dopants have been introduced in PANi chains including hydrochloric acid (HCl), sulfuric acid (H_2_SO_4_), and organic phosphonic acids.^11–15^ Among these, phytic acid (PA) has recently gained attention. PA is a naturally occurring organic acid and has proven to be a highly effective dopant, enhancing both the conductivity and stability of PANi.^16,17^

Unlike monoprotic or simple diprotic acids, PA is a hexaphosphoric acid containing six phosphate groups arranged around an inositol ring. Each PA molecule with its six phosphate groups can release multiple protons and interact with the PANi backbone, as shown in Figure 1. These interactions bridge conductive domains, enhance charge delocalization, and facilitate long-range charge transport.^18,19^ Moreover, the unique multidentate structure of PA offers several advantages beyond simple proton doping. The multiple phosphate groups can form electrostatic interactions and hydrogen bonds with protonated imine sites on adjacent polymer chains, potentially creating interchain bridges that enhance charge delocalization.^20,21^ One aim of the present work is to investigate the charge dynamics in PANi materials. Understanding the dynamics of photogenerated charge carriers in conducting polymers is crucial for optimizing their performance in optoelectronic applications such as organic photovoltaics, photodetectors, and photocatalysts. Charge dynamics are typically taking place in the femto- to nanosecond time scales and requires advance spectroscopic techniques, where light pulses are used to disturb the material’s electronic configuration and monitor its relaxation back to equilibrium.

**Figure 1:**
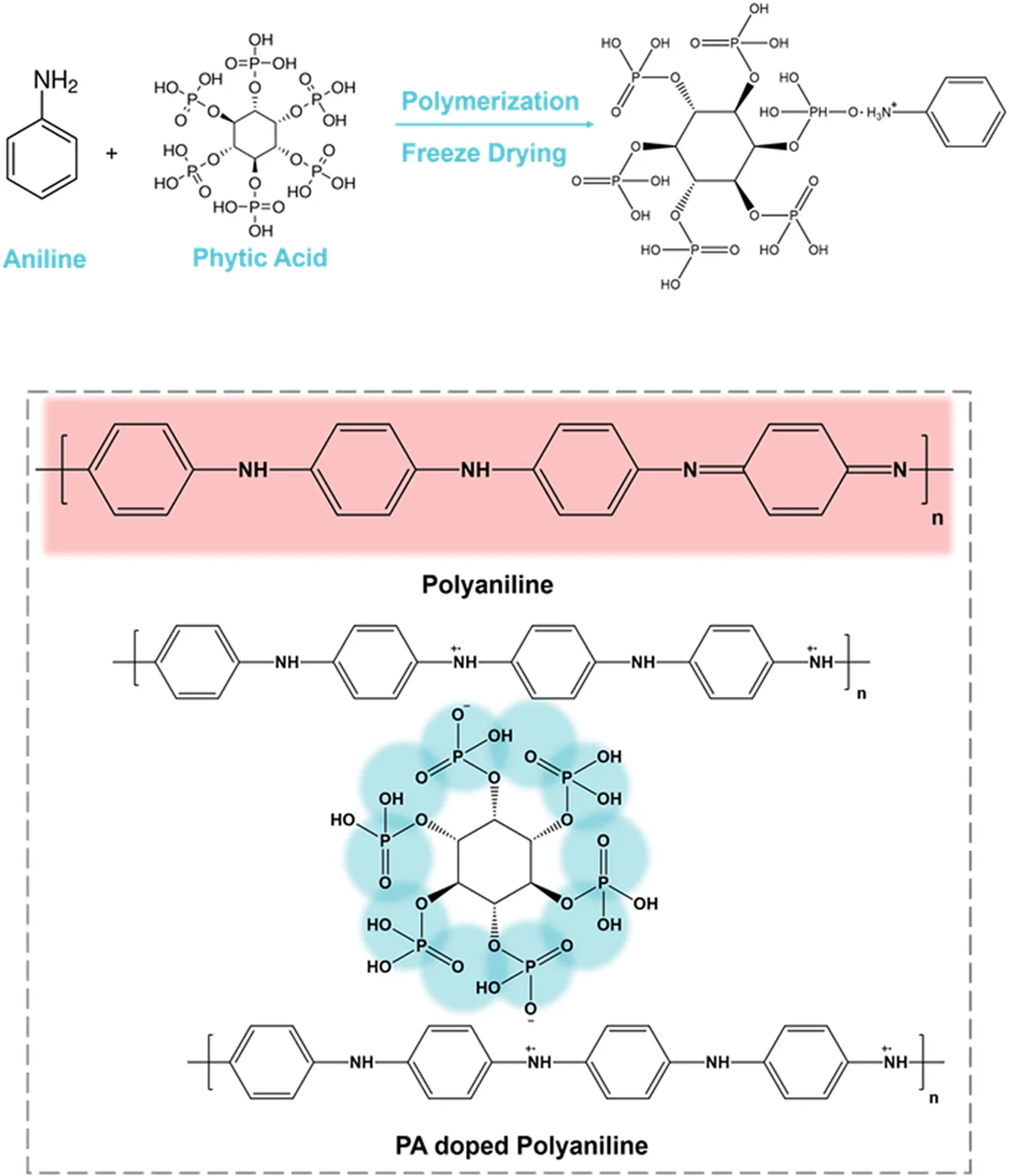
Aniline-phytic acid complex formation (with only one aniline molecule shown complexing to an individual phytic acid for clarity). This is followed by oxidative polymerization, PANi cross-linked by phytic acid. Since each phytic acid molecule can complex with up to six aniline monomers, it acts as both a cross-linker and a proton dopant, facilitating the direct formation of the three-dimensional PANi hydrogel network. For clarity, only a segment of the two-dimensional network building block within the three-dimensional aerogel structure is depicted.

Ultrafast transient absorption spectroscopy (TAS) is a powerful technique for directly probing charge carrier generation, relaxation, and recombination on femtosecond-to-nanosecond time scales. In TAS, a pump laser pulse photoexcites the material to higher energy states, and a probe laser beam, arriving after a controlled time delay, is then used to monitor the lifetimes and dynamics of these excited states.^22^

Despite the importance of PANi and the growing interest in PA as a dopant, to the best of our knowledge, limited studies have explored the ultrafast spectroscopy of PANi. In one study, Kim et al. investigated the relaxation dynamics of photoexcited carriers in primary and secondary doped PANi solutions using pump–probe spectroscopy, revealing subpicosecond to picosecond relaxation processes.^23^ Additionally, Menšík et al. employed femtosecond transient absorption to observe ultrafast dynamics of excited states, charge transfer states, and polaron states in the emeraldine salt form of PANi in solution, providing theoretical descriptions of these processes.^24^ However, these studies focused on conventional dopants, whereas the ultrafast photophysics of PA doped PANi remains comparatively unexplored.

In this study, we investigate the excited-state dynamics and polaron behavior of phytic acid-doped polyaniline nanofibers (PA/PANi) using ultrafast TAS. PA/PANi was synthesized via one-step template-free oxidative polymerization and characterized using X-ray diffraction (XRD), Fourier-transform infrared spectroscopy (FTIR), X-ray photoelectron spectroscopy (XPS), and scanning electron microscopy (SEM) to confirm successful doping and elucidate the material’s structural and morphological properties.

The photophysical properties of PA/PANi were systematically compared with those of commercial pristine (undoped) PANi using steady-state ultraviolet–visible (UV–vis) absorption spectroscopy and femtosecond TAS with multiple pump wavelengths (300, 400, and 600 nm). Our results reveal enhanced polaron lifetimes, indicating the formation of long-lived, delocalized polaronic species. We attribute these observations to PA-mediated interchain coupling that facilitates charge delocalization across polymer chains. These findings provide fundamental insights into the photophysics of PA-doped conducting polymers and highlight their potential for applications requiring efficient charge separation and transport, such as organic electronics and energy storage devices.

## 2 Experimental Section

### 2.1 Materials

Aniline and phytic acid aqueous solution (w/w: 50%) were obtained from Alfa Aesar. ammonium persulfate was purchased from Sigma-Aldrich. Pristine polyaniline was also purchased from Alfa Aesar to compare the ultrafast dynamics of doped samples. All the chemicals were used without any further modification.

### 2.2 Synthesis of Phytic Acid-Doped PANi Aerogels

PANi aerogels were synthesized using a template-free methodology previously reported in the literature and in our prior work,^25,26^ wherein oxidative polymerization occurs in the presence of phytic acid (16% by wt diluted in H_2_O working solution from the 50% stock) and aniline monomers (Figure 1). Phytic acid acts both as the doping agent as well as the surfactant in the reaction. The oxidative polymerization occurs between aniline and phytic acid in the presence of an oxidizing agent, ammonium persulfate.

To synthesize PA/PANi, 5 mL of aniline was mixed with 20 mL of phytic acid, maintaining a 1:4 ratio (by volume). Separately, 1.96 g of ammonium persulfate was dissolved in 10 mL of water. Both solutions were cooled down to 4 °C before being combined and left unstirred at room temperature for 60 min. The gelation period was approximately 3 min. The hydrogels were then immersed in 2 L deionized water for 48 h. The hydrogels were subsequently washed using a vacuum filtration setup to remove any unreacted material. The product was freeze-dried to obtain ∼200 mg aerogel.

### 2.3 Physical Characterization

X-ray powder diffraction patterns were collected on a flat silicon plate using a Bruker-AXS D8 diffractometer using Cu Kα (λ = 1.5418 Å) radiation and a LynxEye position sensitive detector in Bragg–Brentano parafocusing geometry. Sample was scanned from 10 to 50°. Scanning electron microscopy (SEM, Hitachi S-5200, HD-2300A) was employed to determine the morphology of the synthesized sample. The specific surface area was calculated by the Brunner Emmet Teller (BET) nitrogen adsorption–desorption method (BELSORP-mini, BEL Japan, Inc.). The X-ray photoelectron spectroscopy (XPS) analysis was carried out using a Kratos Supra instrument with a monochromated aluminum source. The UV–vis absorption measurements were taken using an Agilent Cary 60 UV–vis spectrometer.

TAS experiments were performed in the Lord Porter Ultrafast Laser Laboratory (ULS), at the University of Sheffield, using a commercial Helios system (HE-VIS-NIR-3200, Ultrafast Systems). A layout of the TAS experimental setup is provided in Figure S1. The TAS module was seeded by a regenerative amplifier (Spitfire ACE PA-40, Spectra-Physics), which provides 800 nm pulses (40 fs FWHM, 10 kHz, 1.2 mJ). The 400 nm pump pulses (2.5 kHz, 0.2 μJ) were generated through frequency doubling of the amplifier’s output. Moreover, narrowband pump pulses at 300 and 600 nm were generated using a TOPAS Prime optical parametric amplifier (Spectra-Physics), which has a tunable output range of 290–1600 nm. The white light probe continuum (440–700 nm) was generated by focusing a portion of the fundamental onto a sapphire crystal. The intensity of the probe light transmitted through the sample was measured using a CMOS camera, with a resolution of 1.5 nm. Before the generation of the white light, the 800 nm pulses were passed through a computer-controlled optical delay line (DDS300, Thorlabs), which provides up to 7 ns of pump–probe delay. The raw pump–probe data were then preprocessed using Surface Xplorer (Ultrafast Systems) before being analyzed using Origin 2021.

## 3 Results and Discussion

The PA/PANi sample was subjected to different characterization techniques to determine the composition and morphological characteristics. The XRD pattern for PA/PANi aerogel is given in Figure 2a. The XRD pattern of PA/PANi shows three broad peaks, characteristic of the semicrystalline emeraldine salt form of PANi. The peak near 15° is often associated with the (011) plane and reflects periodicity along the polymer backbone. The feature at 2θ ≈ 20° corresponds to the (020) plane, while the more pronounced peak at 25.4° is typically linked to the (200) reflection, attributed to π–π stacking between PANi chains. These assignments are consistent with previous reports.^27,28^ The diffraction peaks at specific 2θ values are consistent with the well-known crystal structure of emeraldine PANi. From the above results, it can be inferred that oxidized PANi acquires an ordered structure with alternating benzenoid (fully aromatic) and quinoid (partially oxidized) rings along the polymer chain. These rings are connected by nitrogen atoms, which contribute to PANi’s unique electronic properties. The crystal structure of emeraldine PANi typically exhibits a high degree of planarity and conjugation along the polymer backbone, facilitating efficient charge transport. For comparison, the XRD pattern of commercial pristine PANi (emeraldine base) was also measured and is shown in Figure S2a. The pristine sample exhibits a single broad, poorly resolved diffraction feature centered around 20–25°, characteristic of the largely amorphous or poorly ordered structure of undoped PANi. In contrast, the more distinct features observed in PA/PANi suggest that phytic acid doping enhances structural organization, consistent with its proposed role in bridging and organizing polymer chains.

**Figure 2:**
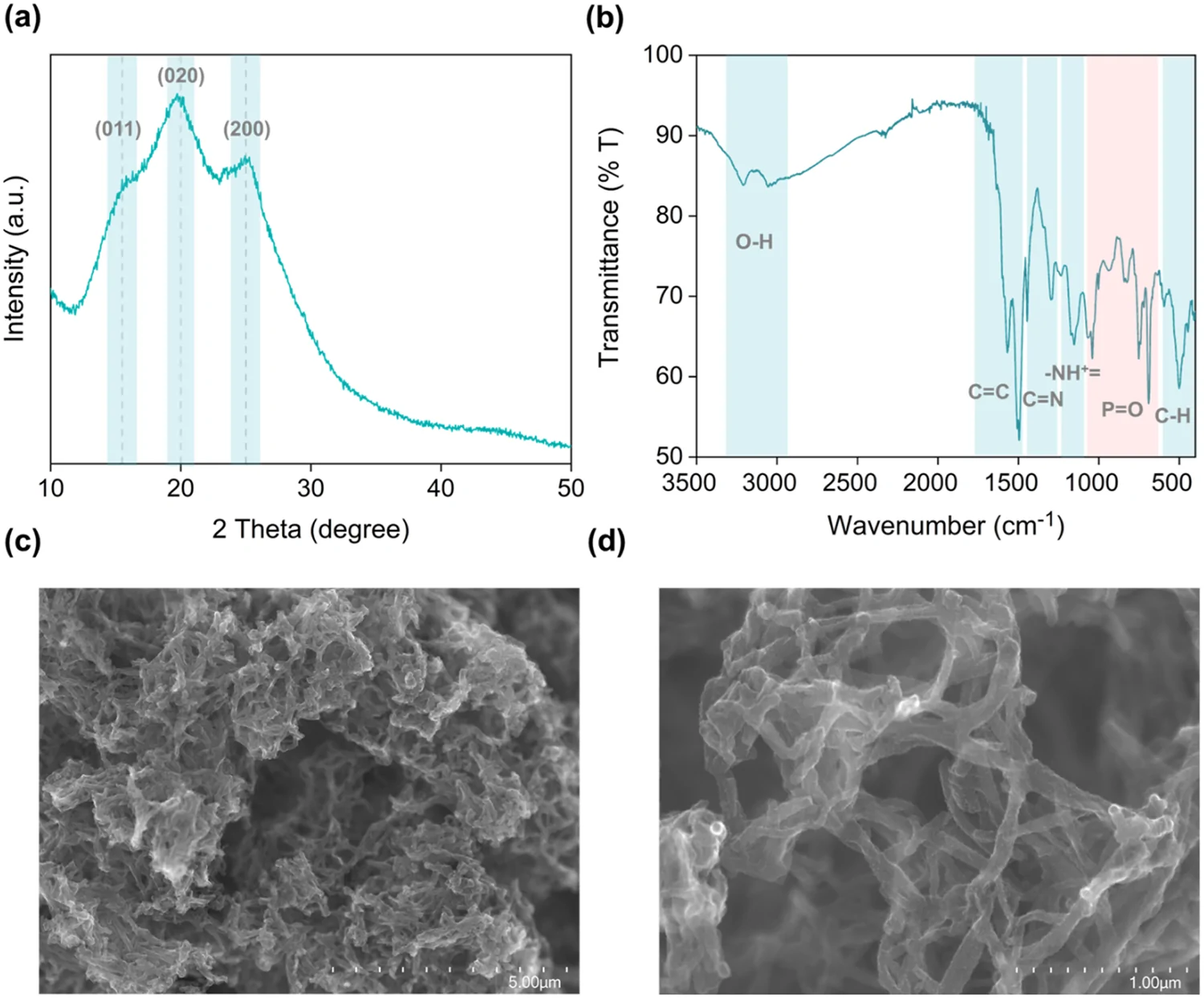
(a) XRD spectra (b) FTIR spectra, and (c, d) Scanning electron microscopy images of the same PA/PANi aerogel fibers at different resolutions. Significant peaks in the XRD and FTIR spectra are highlighted by blue. The P=O FTIR peak is highlighted in pink because it corresponds to the characteristic vibration of the phosphate group from phytic acid, confirming its incorporation into the PANi matrix.

To further investigate the structure and protonation of PANi, FTIR spectroscopy was performed on the PA/PANi aerogel. In the FTIR spectrum of the PA/PANi aerogel (Figure 2b), characteristic bands at 1567 and 1485 cm^–1^ correspond to the C=C stretching vibrations in quinoid and benzene rings, respectively. The bands at 1302 and 1244 cm^–1^ are associated with π-electron delocalization due to protonation and C–N stretching vibrations, as well as the stretching mode of the C–N^+^(polaron structure formed during acid doping of the emeraldine salt form of PANi).^29^ Peaks at 1060, 943, and 878 cm^–1^, assigned to the vibrations of the P=O phosphate group, suggest the presence of phytic acid. These findings indicate successful protonation of PANi by phytic acid following polymerization.^30^ Moreover, to verify these assignments and confirm successful protonation, FTIR spectroscopy was also performed on commercial pristine PANi (emeraldine base), shown in Figure S2b. The pristine sample exhibits the characteristic quinoid and benzenoid bands (1567, 1485 cm^–1^) but shows significantly weaker or absent features in the 1302 and 1244 cm^–1^ region, and completely lacks the PA-specific phosphate vibrations (1060, 943, 878 cm^–1^). This comparison confirms that PA/PANi has been successfully converted to the protonated emeraldine salt form with incorporated PA, whereas the commercial sample remains in the undoped emeraldine base state.

SEM was performed to determine the morphology and microstructure of synthesized PA/PANi aerogels. The material showed the formation of well-established interconnected three-dimensional fiber-like morphology in PA/PANi aerogels, which were obtained by freeze-drying of hydrogels. The SEM images of PA/PANi aerogels are given in Figure 2c,2d, indicating an rough surface. The fibers have an approximate diameter of 50 to 100 nm.

BET analysis was conducted on the synthesized sample to evaluate the surface area. The specific surface area of the PA/PANi aerogels was measured to be 56 m^2^ g^–1^. Additionally, the XPS survey spectrum (Figure 3d) of the PA/PANi aerogel predominantly shows peaks of carbon (C), nitrogen (N), phosphorus (P), and oxygen (O). XPS reveals that the aerogel contains carbon, nitrogen, phosphorus, and oxygen in atomic ratios of 74.3%, 9.1%, 4.0%, and 12.6%, respectively.

**Figure 3:**
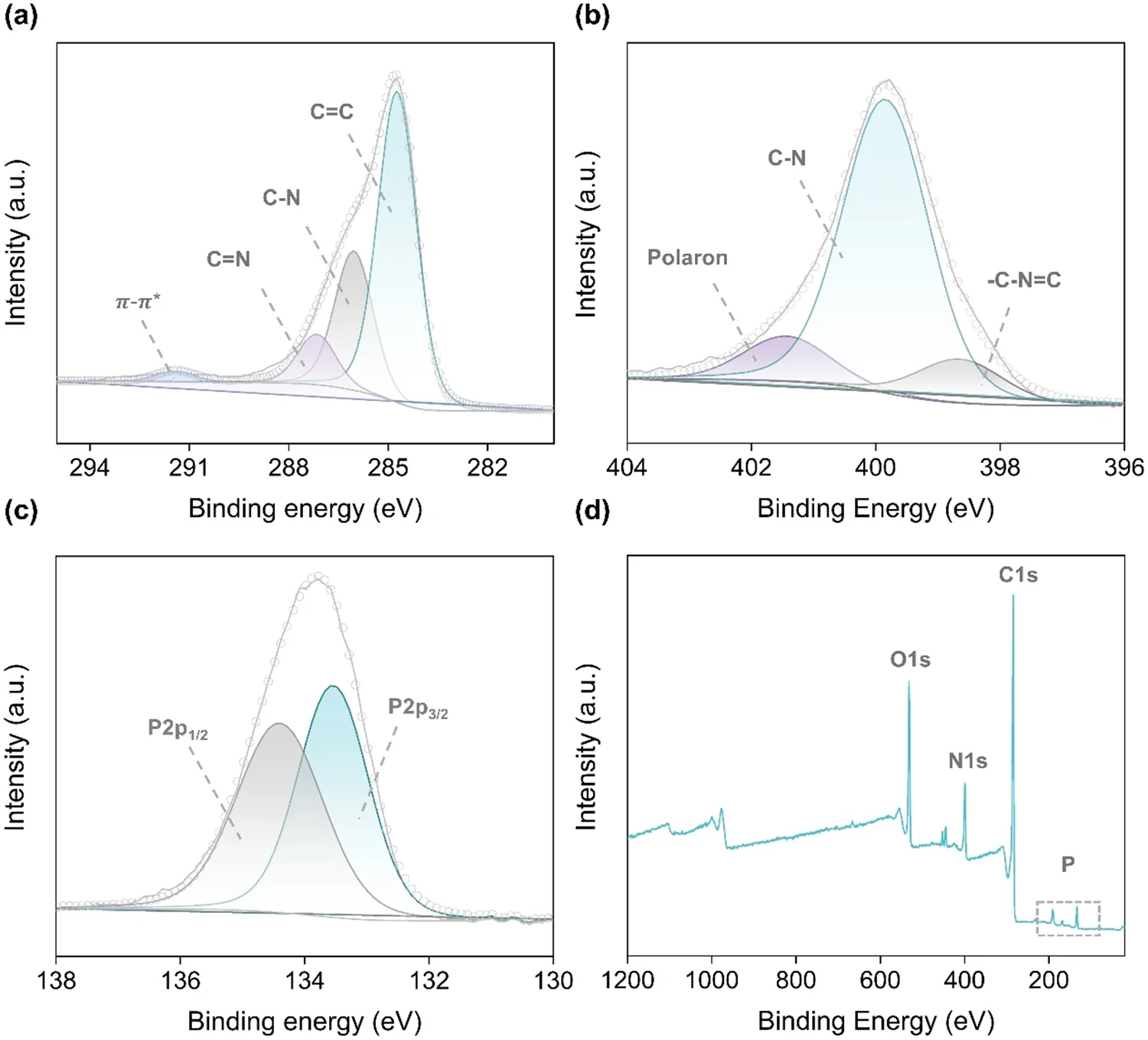
High-resolution spectra of (a) C 1s, (b) N 1s (c) P 2p, and (d) XPS survey spectrum of PA/PANi aerogel.

The C 1s XPS spectrum of PA/PANi is given in Figure 3a. The deconvoluted spectrum depicts four characteristic regions corresponding to the C–C/C=C polymer backbone at 284.6 eV, C–N bonds at 285.8 eV and protonated C=N band at 287.7 eV. Additionally, a π–π* satellite peak is observed at around 291 eV.^31^

The deconvoluted N 1s spectrum of the PA/PANi aerogel (Figure 3b) was fitted with three components. The peak at ∼398.0 eV is assigned to the quinoid imine nitrogen (=N-), while the dominant component at ∼399.5 eV corresponds to the benzenoid amine nitrogen (−NH−). The higher binding-energy feature at ∼401.5 eV is attributed to positively charged nitrogen (N^+^), characteristic of polaronic species in the protonated emeraldine salt, confirming proton doping of PANi by phytic acid.^32^

The P 2p spectrum of the PA/PANi aerogel (Figure 3c) exhibits a spin–orbit-split doublet, with the P 2p_3/2_ and P 2p_1/2_ components centered at ∼133.5 and ∼134.3 eV, respectively. These binding energies are characteristic of pentavalent phosphorus in the phosphate groups of phytic acid. The O 1s signal likewise originates predominantly from these phosphate groups.^33^

The normalized UV–vis spectra of commercial pristine PANi (emeraldine base) and PA-doped PA/PANi in dimethylformamide (DMF) are given in Figure 4. DMF was used as a noninteracting and transparent solvent to facilitate the transient spectroscopic characterization of polyaniline. The UV–vis absorption of PANi depends on several factors such as the doping level, conjugation, and solvent.^34^ The commercial PANi spectrum exhibits two primary absorption bands typical of PANi: one around 350 nm, attributed to the π–π* transition within the benzenoid rings and one band around 600 nm that corresponds to excitonic transition interbond charge transfer between benzenoid to quinoid moieties.^35,36^ On the other hand, the UV–vis spectrum of PA/PANi exhibits three distinct absorption bands within the wavelength ranges of 250–320 nm, 320–450 nm, and 480–750 nm. With exposure to PA, the band centered at around 600 nm shows a significant reduction in intensity, which is common with chemical doping. However, unlike PANi, this band in PA/PANi can be attributed to mixed polaron and interband charge transfer. The band between 250 and 320 nm corresponds to the π–π* transition, arising from the excitation of nitrogen in the benzenoid segments, while the peak at ∼400 nm is assigned to the polaron band to the π*, associated with the protonated imine groups (C=N) in PA/PANi.^37–39^ Moreover, absorption below 270 nm, which is typically associated with transitions in conjugated systems, was excluded due to noise.

**Figure 4:**
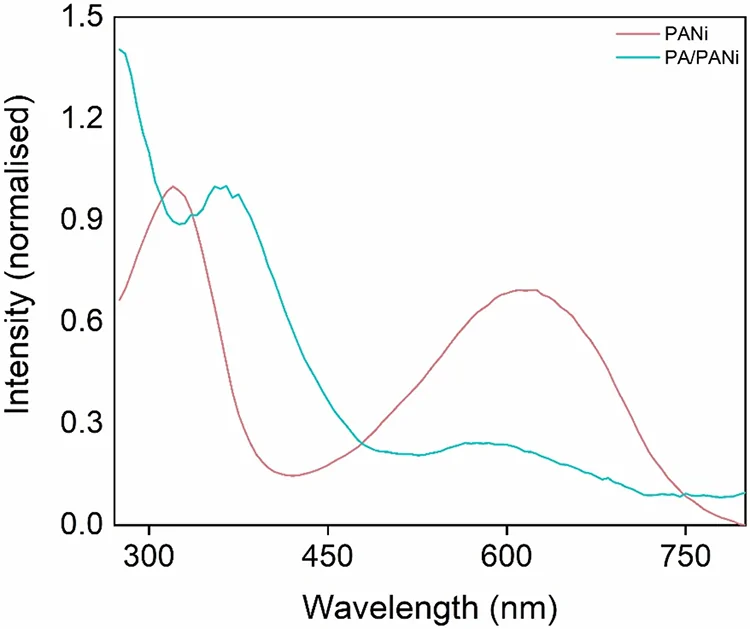
Normalized UV–vis absorption spectra of commercial PANi and PA/PANi in DMF, normalized to the peak at ∼330 nm.

Additionally, to confirm the formation of the emeraldine salt and assess solvent effects, UV–vis spectroscopy of PA/PANi was also performed in aqueous solution (Figure S3). The aqueous spectrum exhibits a characteristic broad absorption extending into the near-infrared region with a feature centered around 800 nm, which is attributed to free carrier (polaron) absorption typical of heavily doped conducting polymers. This feature is less pronounced in the DMF spectrum, likely due to differences in solvent polarity affecting chain conformation and polaron delocalization. The presence of the 800 nm band in water confirms the conductive emeraldine salt form of PA/PANi.

Ultrafast TAS was employed to investigate the dynamics of excited states and charge carriers in commercial PANi and PA/PANi. Initially, the transient absorption dynamics of PA/PANi in DMF were investigated using a 400 nm pump, corresponding to the polaron-π* transition characteristic of protonated PANi. The feature is notably absent in commercial/undoped PANi.

The TA spectra of PA/PANi were recorded at different delay times up to 6 ns (Figure 5a). Following the excitation at 400 nm, the spectra exhibited a strong excited state absorption (ESA) feature spanning 450–650 nm. The ESA is indicative of polaronic transitions within the polymer backbone. Polarons in PA/PANi are charge carriers (typically radical cations) that are coupled with local distortions in the conjugated backbone. The transition, polaron to π*, is photoexcitation of those polaronic species. The presence of ESA in this region suggests active polaronic dynamics in the photoexcited state. Moreover, the intensity of the observed ground-state bleach (GSB) below 450 nm may be reduced due to the predominant ESA signal. The ESA dynamics showed a decay within ∼100 ps, transitioning to a negative signal that persisted throughout the time delay window, suggesting the formation of a long-lived intermediate or the final state, likely due to charge delocalization within the polymer matrix due to the extended conjugation.^40,41^

**Figure 5:**
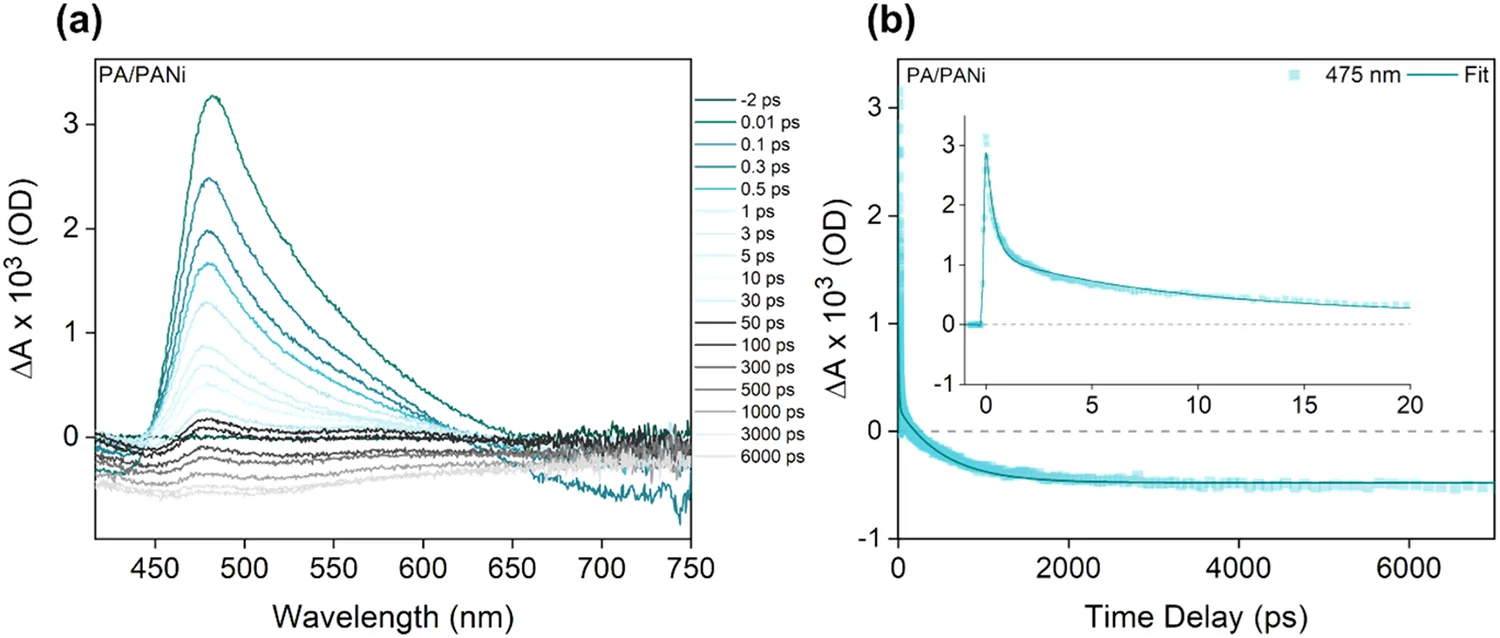
(a) Selected transient spectra PA/PANi in DMF at different delay times (up to 6 ns) following excitation at 400 nm with a laser pulse (b) Transient absorption kinetics of PA/PANi in DMF at 475 nm showing a three-component decay (∼380 fs, ∼8.6 ps, and ∼537 ps) following 400 nm excitation, with an additional long-lived nondecaying component.

Moreover, TAS kinetics analysis at 475 nm, probing the ESA region, reveals a three-component decay process (Figure 5b). The decay dynamics were fitted using a multiexponential model, reflecting the complex relaxation processes in PA/PANi. The initial fast decay of ∼380 fs is attributed to charge carrier relaxation (shown in the inset image of Figure 5b), a transition previously highlighted in studies of chemically doped polymers.^42^ It was followed by a ∼8.6 ps component likely corresponding to structural relaxation around polarons. A slower ∼537 ps component was ascribed to polaron relaxation or equilibration processes, while the nondecaying component indicated the delocalization of residual charge carriers, likely due to the structural changes introduced by PA doping.^25^ This is linked to the enhanced connectivity between conductive domains introduced by PA doping, which is also responsible for the conductive behavior of conjugated polymers.^43^ In a similar study, Ballabio et al. observed that PA doping leads to enhanced charge transport by enabling long-range charge transfer between adjacent conductive domains, which may influence polaron dynamics.^44^ Moreover, to confirm the rise of the signal, the TAS kinetics were compared with the instrument response function (IRF), given in Figure S6.

To compare the ultrafast dynamics of PA/PANi and pristine PANi, both samples were subjected to a 600 nm pump wavelength, corresponding to interband charge transfer between the benzenoid and quinoid moieties of PANi.^45^ However, in PA/PANi this wavelength likely overlaps with protonation or structural changes induced by PA.

Upon excitation, PANi exhibits a broad transient signal in the spectral region of 425–525 nm, characteristic of pristine PANi. This ESA band can be attributed to exciton transitions within the polymer chains (Figure 6a). The signal then decays within 5 ps time due to the relaxation of excited states, indicating that the excited states generated are unstable and undergo fast recombination. Kinetic analysis at 475 nm shows a biexponential decay (Figure 6b), revealing two distinct time constants: a fast decay component of approximately 0.3 ± 0.1 ps and a slower decay component of around 4 ± 0.3 ps. The fast decay likely corresponds to rapid nonradiative relaxation. The second component, with a lifetime of around 4 ps, represents a short-lived excited state.^46^ Beyond that, PANi shows a negligible negative signal, characteristic of long-lived photoinduced charged states.

**Figure 6:**
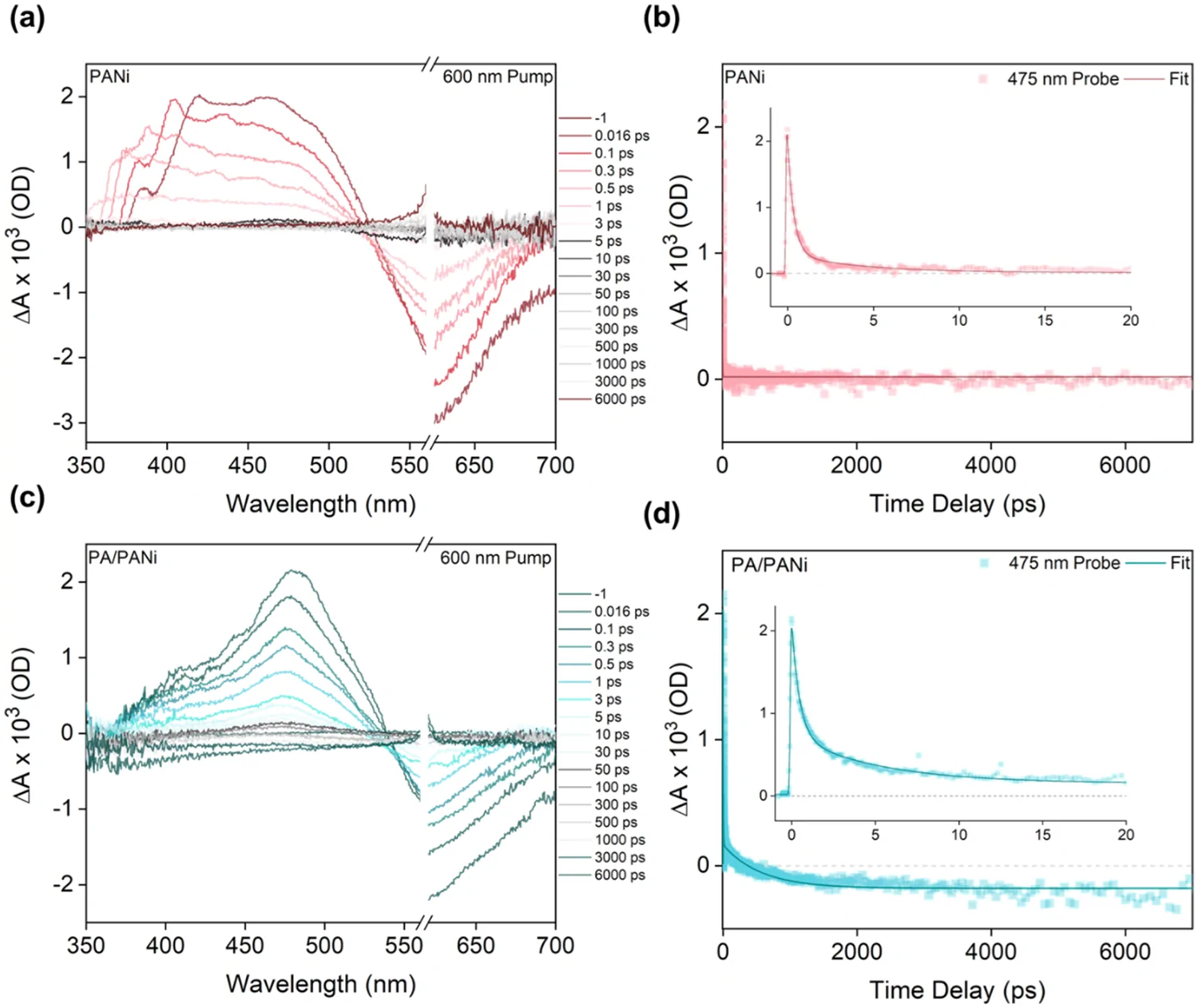
Transient absorption spectra and kinetics following 600 nm excitation. (a) Selected transient spectra of pristine PANi in DMF at different delay times (up to 6 ns). (b) Corresponding transient absorption kinetics of pristine PANi at 475 nm probe wavelength showing biexponential decay (0.3 ± 0.1 ps and 4 ± 0.3 ps). (c) Selected transient spectra of PA/PANi in DMF at different delay times. (d) Corresponding transient absorption kinetics of PA/PANi at 475 nm probe wavelength. The 560–620 nm spectral region has been removed because of pump scatter. A four-component fit yields ∼0.5 ± 0.1 ps, 5 ± 0.2 ps, 568 ± 24 ps, and a nondecaying component that lives beyond 7 ns.

In contrast, PA/PANi shows an ESA signal with reduced absorption below 450 nm (Figure 6c). This spectral difference indicates changes in the oxidation state of the polymer upon doping. To understand the ultrafast dynamics of excited states and charge carriers in PA/PANi, the transient absorption kinetics are compared with undoped PANi at probe wavelengths of 475 nm. The kinetics of PA/PANi exhibited a more complex decay profile (Figure 6d). A four-component fit was required to accurately fit the decay dynamics, with constants of approximately 0.5 ± 0.1 ps, 5 ± 0.2 ps, 568 ± 24 ps, and a longer-lived nondecaying component (longer than our 7 ns experimental window). The first component, however, constitutes more than 80% of the total amplitude. All the lifetimes and their corresponding amplitudes at various pump wavelengths are summarized in Table 1. The multiple decay components suggest a complex relaxation process, possibly involving different charge carriers with varying lifetimes in the doped polymer matrix. Beyond the 300 ps time scale, the PA/PANi displayed a persistent strong negative signal that extended up to 7 ns. Moreover, kinetics analysis of PANi and PA/PANi up to 300 ps following excitation at 600 nm is given in Figure S4 for comparison. The negative signal observed is characteristic of long-lived photoinduced charged states.^23^ The strong and extended presence of this negative transient absorption signal suggests the formation of delocalized polaronic species that could be due to interchain interactions due to PA doping, aligning with the idea that phytic acid bridges between polymer chains and enhances charge delocalization.^47^

**Table 1:** Lifetimes and Corresponding Amplitudes at 475 nm for Different Pump Wavelengths^a^

pump	sample	τ_1_ (ps)	A_1_ (OD)	τ_2_ (ps)	A_2_ (OD)	τ_3_ (ps)	A_3_ (OD)	τ_4_ (ps)	A_4_ (OD)
300 nm	PANi	0.6 ± 0.1	3 ± 0.03	37 ± 4.38	0.18 ± 0.08	>4 ns	0.09 ± 0.007	_	_
	PA/PANi	0.5 ± 0.1	3.75 ± 0.1	10 ± 0.8	0.638 ± 0.02	834 ± 64	0.43 ± 0.01	nd	–0.06 ± 0.01
400 nm	PA/PANi	0.4 ± 0.1	2.43 ± 0.04	8.6 ± 0.321	0.95 ± 0.01	537 ± 19	0.68 ± 0.008	nd	–0.47 ± 0.01
600 nm	PANi	0.4 ± 0.1	2.28 ± 0.04	4 ± 0.36	0.289	_	_	_	_
	PA/PANi	0.5 ± 0.1	1.6 ± 0.02	5 ± 0.2	0.58 ± 0.25	568 ± 24	0.34 ± 0.005	nd	–0.19 ± 0.007

a “nd” stands for “non-decaying”, i.e., signals that do not decay within the measured time window.

Since distinct differences were observed between PA/PANi when excited at 600 nm, the TAS behavior of PANi and PA/PANi dissolved in DMF was also observed following excitation at 300 nm. The signal corresponds to the π-π* transition of the benzenoid unit (S_0_ → S_1_).^48^

Upon excitation, rapid ESA from the S_1_ state of PANi to higher electronic states occurs, appearing as a broad transient signal in the early spectra (Figures 7a). The signal of PANi ranges from 360 nm to approximately 520 nm and exhibits a characteristic decay pattern. The transient absorption kinetics at 475 nm (Figure 7b) reveal a single-exponential rise with a time constant of 0.63 ± 0.01 ps, followed by a biexponential decay with time constants of 37 ± 4 ps and a long component >4 ns. The ESA signal mostly decays in the first ∼50 ps due to depopulation of the S_1_ state.

**Figure 7:**
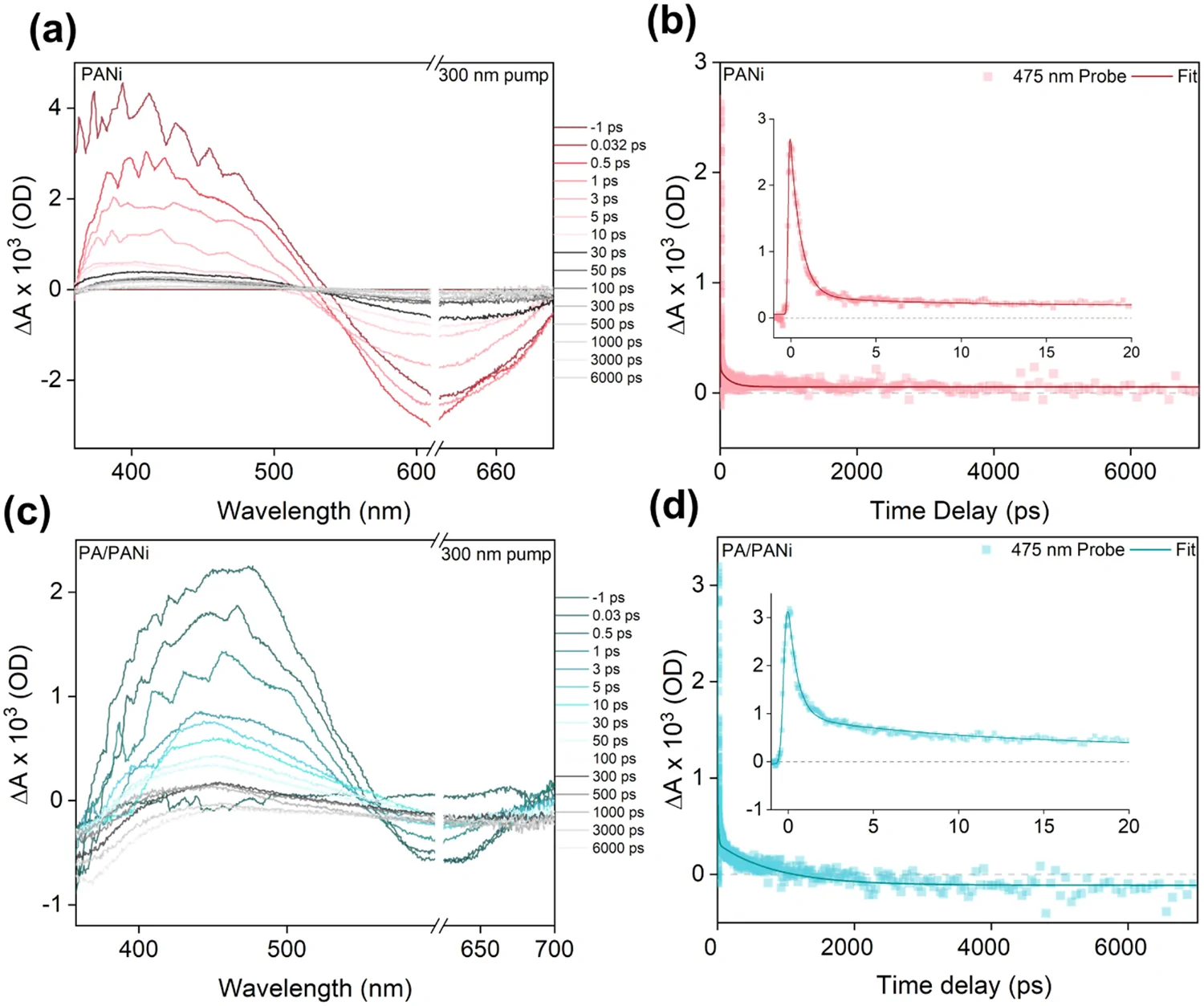
(a) PANi in DMF at different delay times (up to 6 ns) following excitation at 300 nm, with the 610–620 nm region cut to eliminate pump scatter. (b) Transient absorption kinetics of PANi in DMF at 475 nm probe wavelength following 300 nm excitation. The kinetics show a single-exponential rise (0.6 ± 0.1 ps) followed by a biexponential decay (37 ± 4 ps and >4 ns). (c) Transient absorption spectra of PA/PANi in DMF at different delay times (up to 6 ns) following excitation at 300 nm, with the 610–620 nm region removed to eliminate pump scatter. (d) Transient absorption kinetics of PA/PANi in DMF following excitation at 300 nm at a probe wavelength of 475 nm, fitted with a multicomponent model showing a rise time of 0.5 ± 0.1 ps, decay components of 10 ± 0.8 ps and 834 ± 64 ps, and a long-lived nondecaying signal.

In contrast, PA/PANi exhibits a broader ESA signal, ranging from 360 nm to approximately 560 nm, which persists for ∼100 ps. Moreover, the kinetics analysis of PA/PANi at 475 nm (Figure 7d) shows distinct photophysical behavior compared to PANi. The rise time is in the same order of magnitude (0.5 ± 0.1 ps), but the decay time constants alter significantly with time constants of 10 ± 0.8 ps, 834 ± 64 ps, as well as an additional nondecaying negative component. Furthermore, in PA/PANi, the contribution of the second component to the overall signal, compared to that of the first short-lived component, is larger than in PANi (Table 1), meaning that the associated process is more prominent in PA/PANi. The negative signal in PA/PANi at 475 nm probe wavelength at later time likely reflects long-lived delocalized polaronic species stabilized by PA-induced structural modifications and interchain interactions.^49,50^ Detailed kinetic analysis of PANi and PA/PANi at 475 nm probe up to 300 ps is given in Figure S5.

To provide complementary evidence for charge transport, the effective in-plane electrical conductivity of the phytic acid-doped polyaniline-coated electrode was measured using a four-point probe method. The measured effective conductivity, experimental details, and data analysis are provided in the Supporting Information.

## 4 Discussion

The spectral differences between PANi and PA/PANi at different pump wavelengths, particularly in the polaron band, confirm that PA doping alters the electronic structure of PANi. It is attributed to enhanced charge delocalization or modification of the polymer chain conformation. This is due to the ability of PA to coordinate with multiple PANi chains bridging adjacent conductive domains and enhancing a long-range charge transport as studied by Ballabio et al.^44,51^

Moreover, the spectral shift between the two indicates changes in the oxidation state of the polymer upon doping. The kinetics analysis of two at 475 nm further revealed distinct differences in dynamics. As a strong proton donor, PA increases concentration of charge carriers and facilitates their delocalization along the polymer backbone.^52,53^ Additionally, this could be due to the interaction between phytic acid and the nitrogen atoms in polyaniline due to the formation of strong hydrogen bonds and electrostatic interactions within the polymer structure.^50^ Such electronic modifications lead to a more robust charge carrier lattice, which explains the extended positive and negative signals observed in the TAS spectra of the doped sample. Moreover, the rapid decay of signal in pristine PANi indicates that in the absence of a dopant, the excited states are inherently unstable and prone to recombination on a very short time scale. This is in line with Xia et al.’s findings, where they showed that the polaronic behavior of protonated polyaniline is influenced by both solvent and dopant, affecting whether polarons are localized or delocalized.^54^ These characteristics position PA/PANi as a promising candidate for applications such as optoelectronic devices, energy storage systems, and sensors, where stable and efficient charge transport is crucial.

## 5 Conclusion

In this work, we investigated the photophysical properties of phytic acid-doped polyaniline (PA/PANi) synthesized via a one-step, facile oxidative polymerization process. Structural and compositional analysis confirmed the successful synthesis and revealed a nanofibrous morphology in PA/PANi. The photophysical properties of PA/PANi were studied using transient absorption spectroscopy (TAS). TAS results were compared with the undoped commercial PANi sample. Compared to undoped PANi, the TAS of PA/PANi depicted enhanced charge delocalization and prolonged excited-state lifetimes across multiple pump wavelengths (300, 400, and 600 nm), indicating improved charge mobility dynamics. The extended charge carrier lifetimes and delocalized excited states indicates more efficient charge mobility in the doped system. Overall, these findings demonstrate that phytic acid doping can tune the charge transport properties of conducting polymers, enabling their application in advanced optoelectronic and energy storage devices.

## Supplementary Material



## Notes

TAS beam path and component layout; XRD and FTIR spectra of pristine PANi; UV–vis spectrum of PA/PANi in water; TAS kinetics comparisons following 600 and 300 nm excitation; instrument response function comparison; four-point probe conductivity method, sample preparation, and test device schematic (PDF)
