# Supplementary material for: Enhanced Polaron Delocalization in Phytic Acid-Doped Polyaniline Nanofibers
Source: Chem Mater. 2026 Aug 3;38(16):8157–66. doi: 10.1021/acs.chemmater.6c00240 (PMC13523630; doi:10.1021/acs.chemmater.6c00240)
Supplement: Supplementary file 1 [file cm6c00240_si_001.pdf]

Supplementary Information

Enhanced Polaron Delocalization in Phytic Acid-Doped Polyaniline Nanofibers

Laraib Nisar[1], Martin V. Appleby[1,2], Abeera Hassan[3], Benedict J Smith[1], Dimitri Chekulaev[1], Qizhi Ba[4], Mohamed Pourkashanian[5], Mohammed S Ismail[6*], Adrien A. P. Chauvet[1*]

[1]Dainton Building, Department of Chemistry, The University of Sheffield, Brook Hill, Sheffield, South Yorkshire S3 7HF, United Kingdom.

[2]Paul Scherrer Institut, Forschungsstrasse 111, Villigen PSI, 5232 Switzerland.

[3]Department of Chemistry, 324 Brook Street, Brown University, Providence, Rhode Island 02912, United States.

[4]Energy 2050, Department of Mechanical Engineering, Faculty of Engineering, University of Sheffield, Sheffield S3 7RD, United Kingdom.

[5]Translational Energy Research Centre, The University of Sheffield, Sheffield S9 1ZA, United Kingdom.

[6]School of Engineering, University of Hull, Hull HU6 7RX, United Kingdom.

To whom correspondence should be addressed.
*Adrien Chauvet a.chauvet@sheffield.ac.uk
*Mohammed S Ismail m.s.ismail@hull.ac.uk

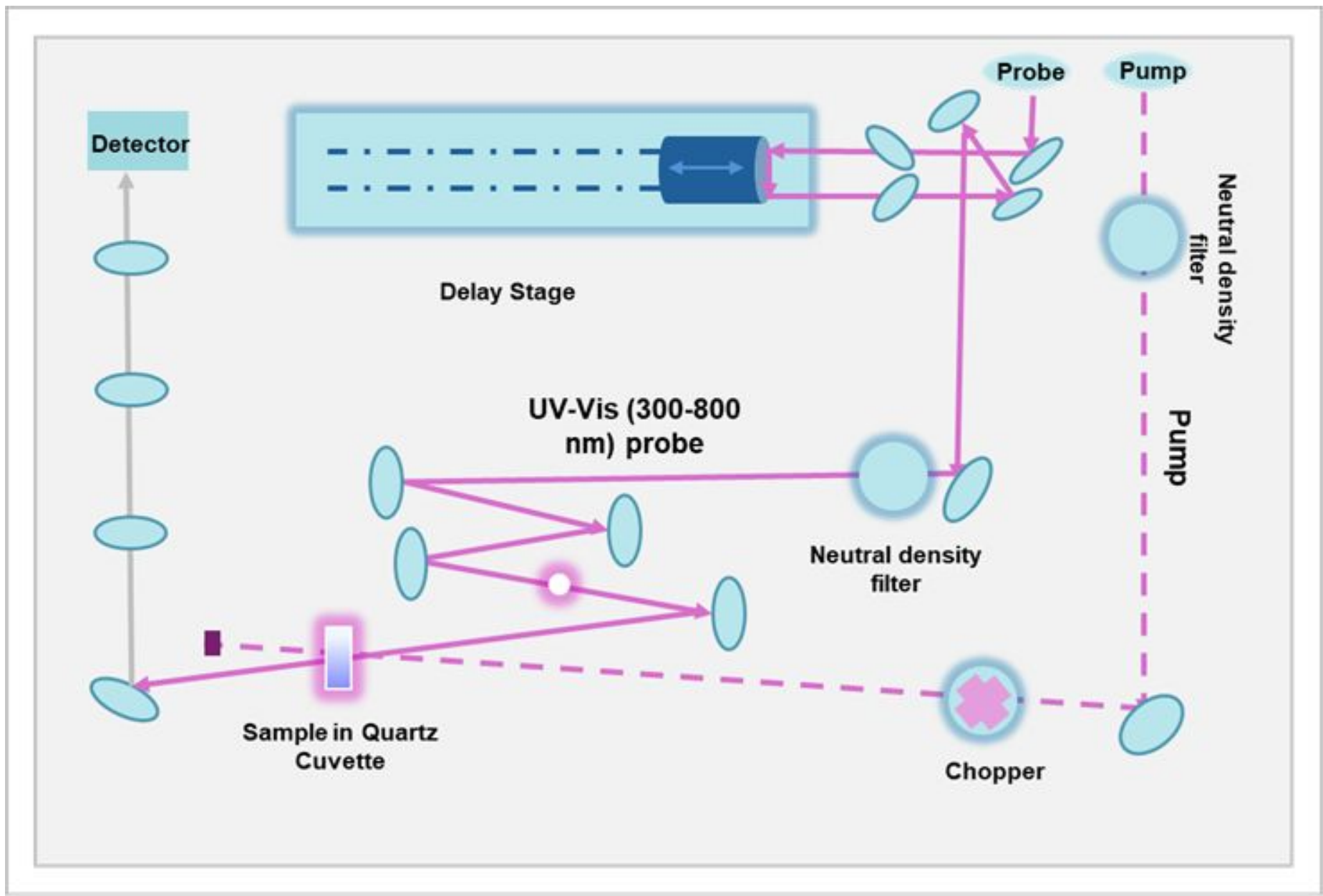

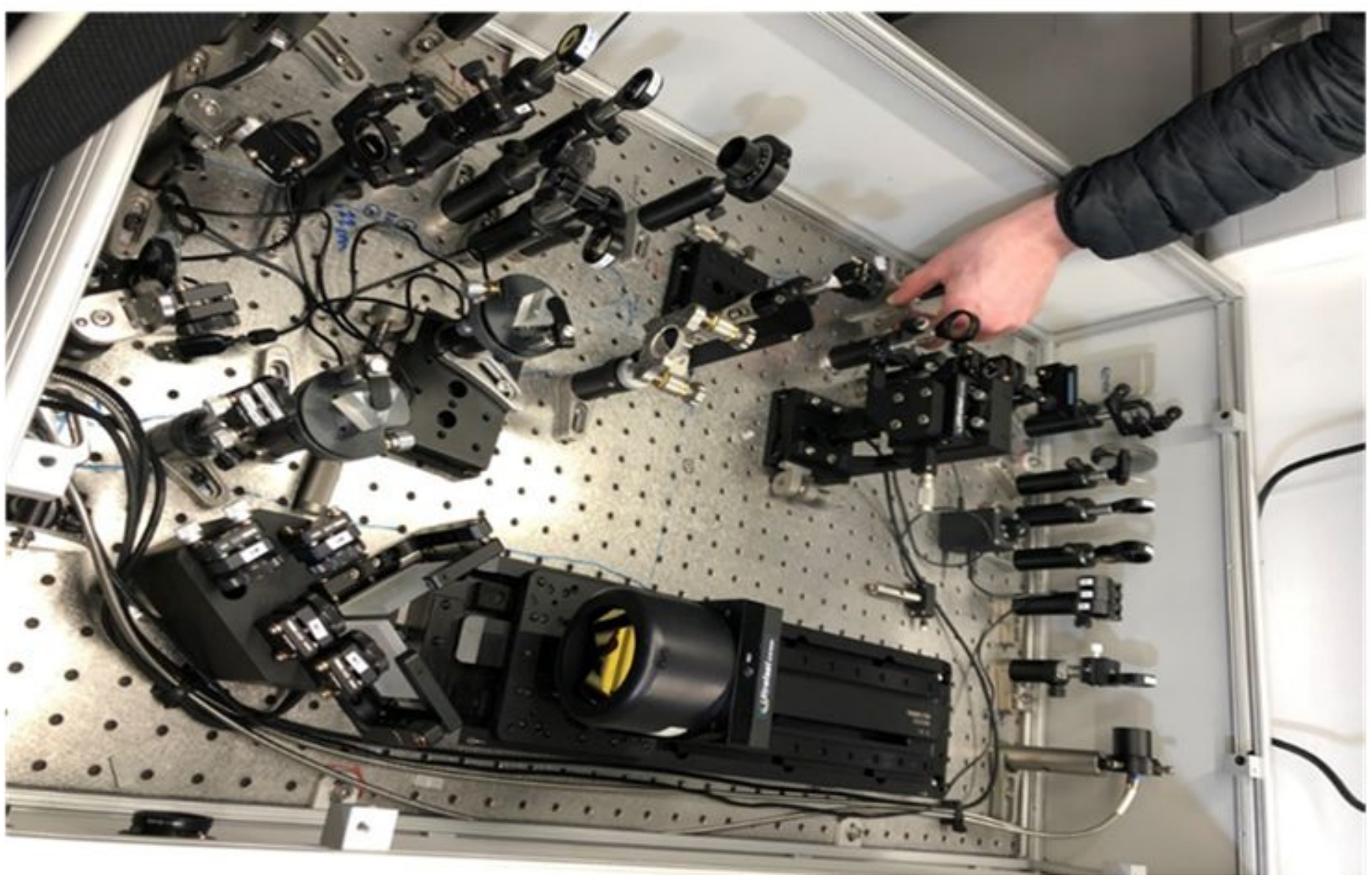

Figure S 1: Transient absorption component layout and beam path. Oval shaped optical components correspond to alignment mirrors and lenses.

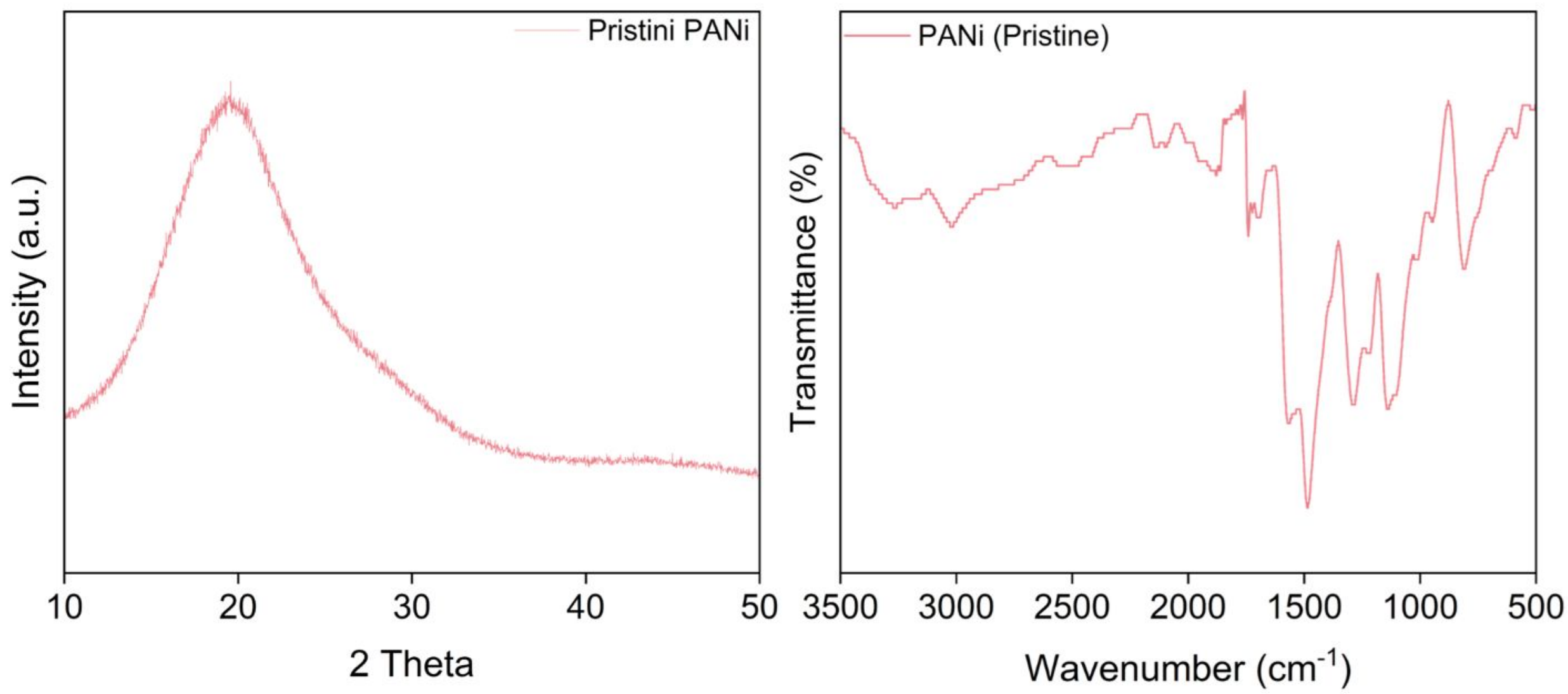

Figure S 2: **(a)** XRD spectra **(b)** FTIR spectra of pristine PANi

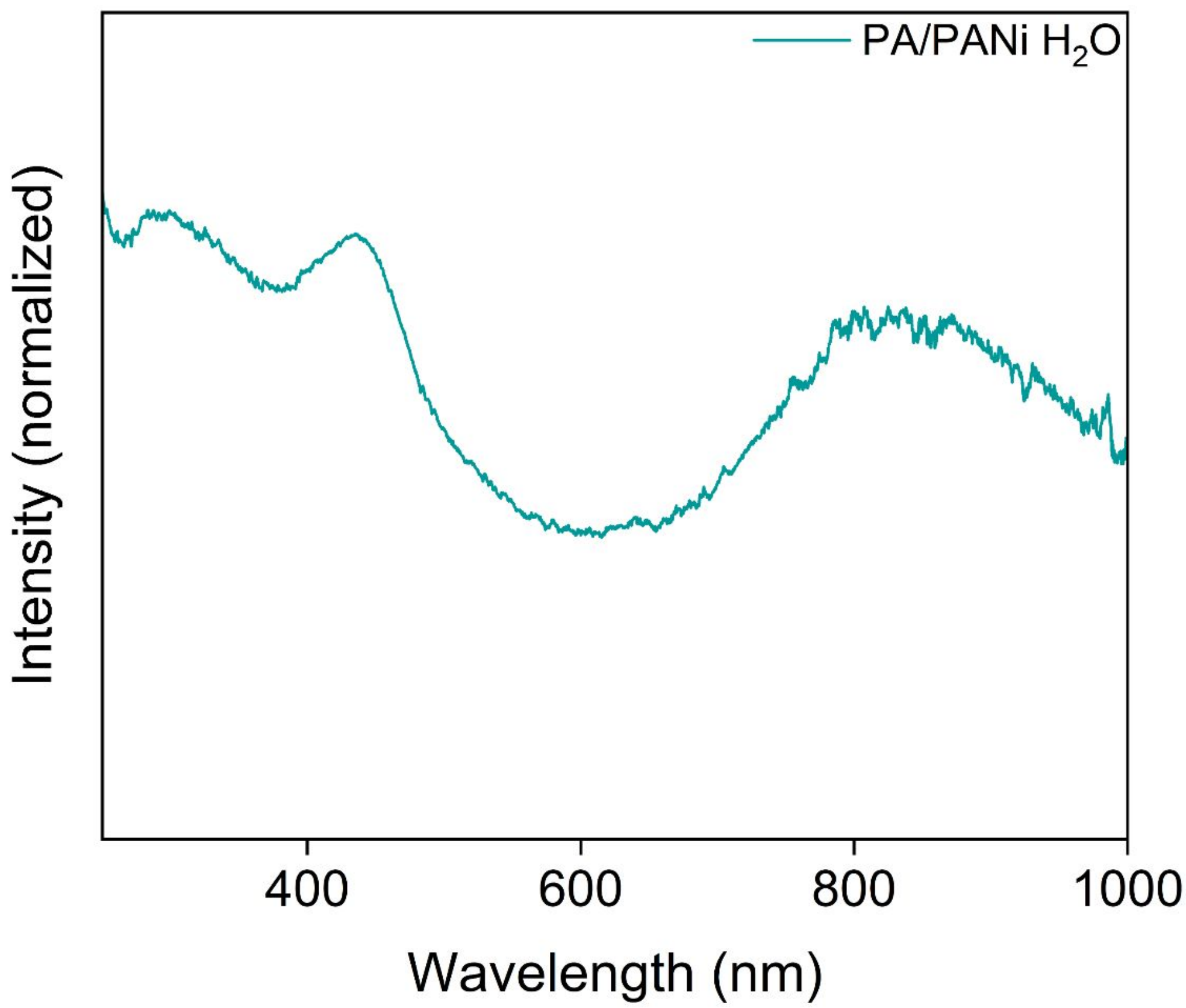

Figure S 3: Normalised UV-Vis absorption spectra of PA/PANi in water.

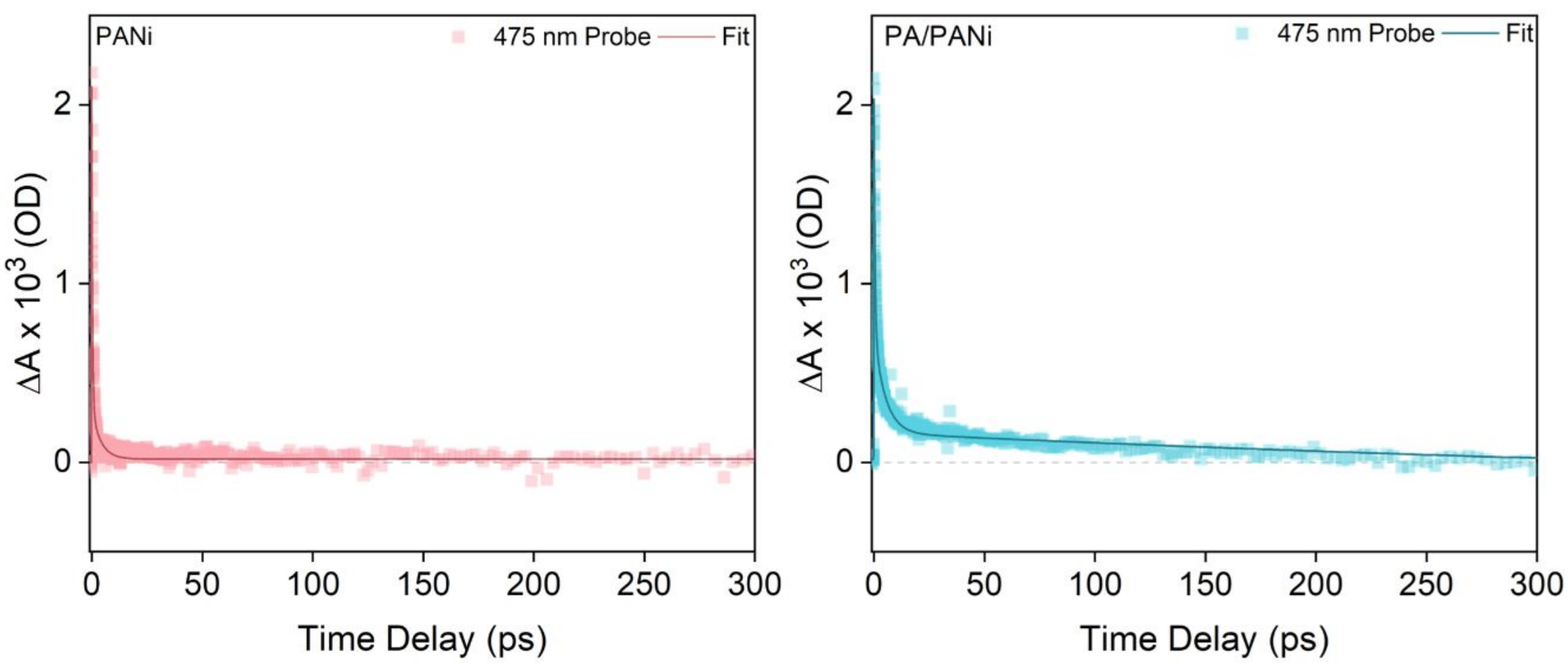

Figure S 4: Kinetics of PANi and PA/PANi at 475 nm up to 300 ps following excitation at 600 nm.

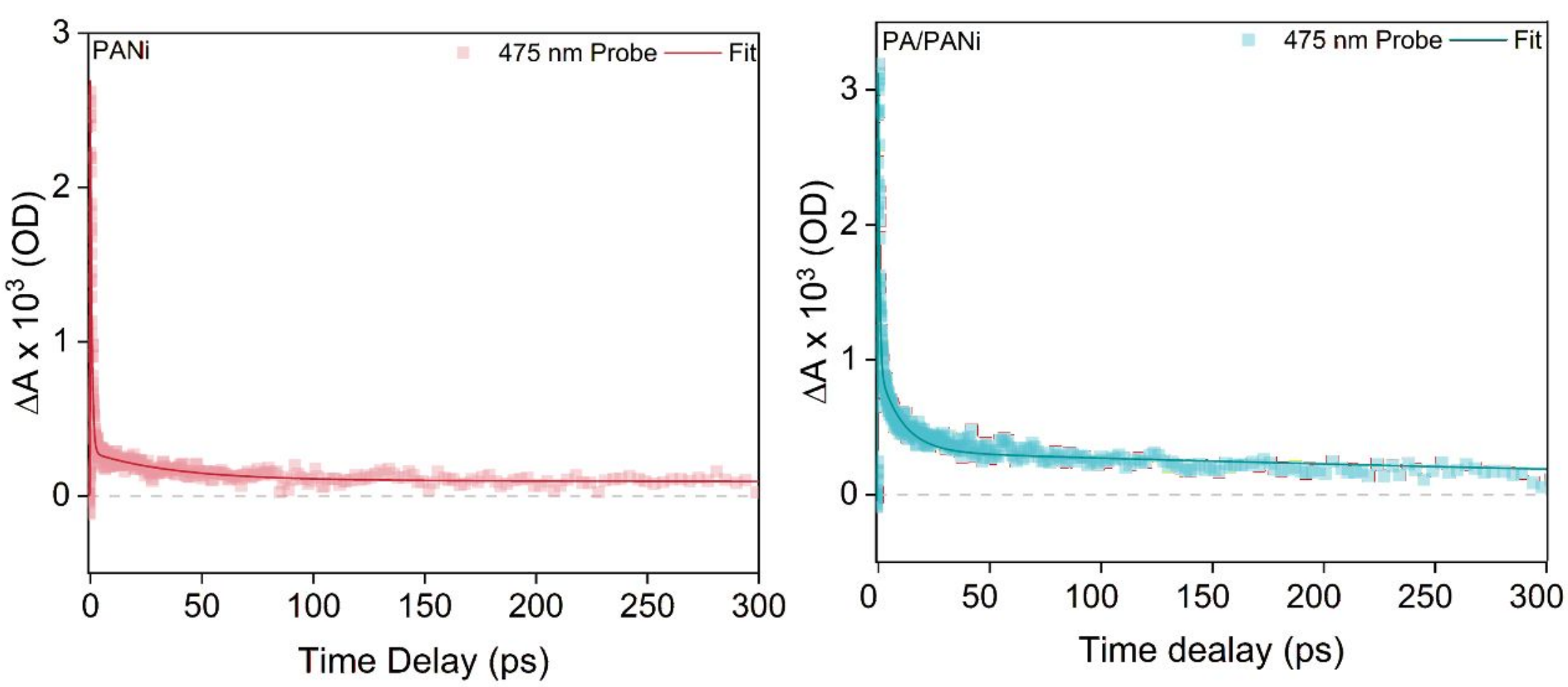

Figure S 5: Kinetics of PANi and PA/PANi at 475 nm up to 300 ps following excitation at 300 nm.

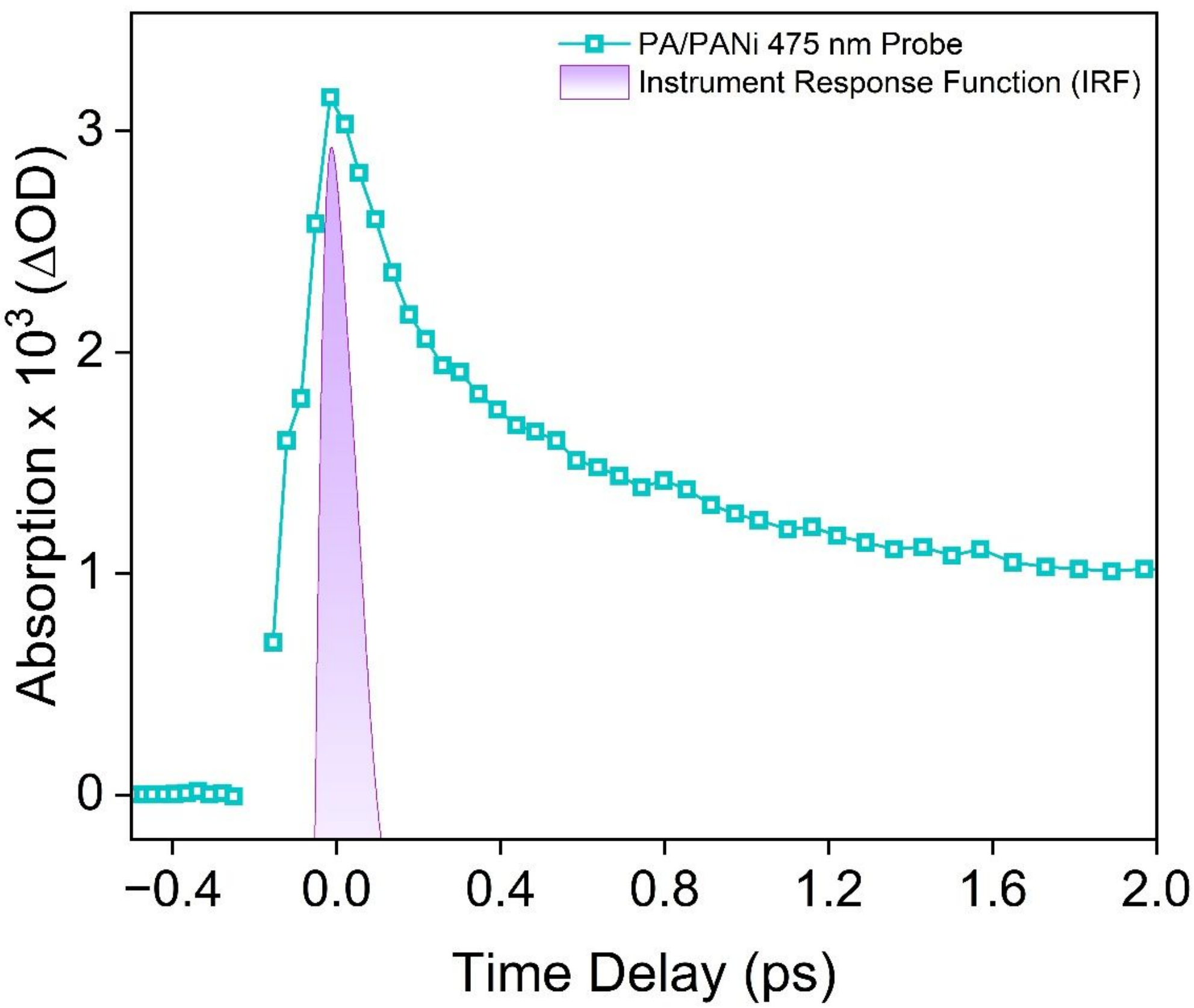

Figure S 6: Comparison of TAS kinetics with the instrument response function (IRF), confirming that the observed signal rise is not limited by the instrument response.

Effective In-Plane Electrical Conductivity Measurement

Sample Preparation

The effective in-plane electrical conductivity was measured for phytic acid-doped polyaniline-coated electrodes. The coating ink was prepared by dispersing the active material with Triton X-100 (Sigma-Aldrich, UK) as the surfactant and methyl cellulose (Sigma-Aldrich, UK) as the binder in deionized water. The mixture was stirred at 600 rpm for 10 min and subsequently doctor-blade coated onto Toray TGP-H-60 carbon paper (10 wt% wet-proofed, Fuel Cell Earth, USA). The coated electrodes were dried at 80 °C to remove residual solvent prior to electrical characterization.

Four-Point Probe Conductivity Measurement

The effective in-plane electrical conductivity of the coated electrodes was measured using a four-point probe configuration based on the method reported by Ismail *et al.*[1] Four equally spaced probes (20 mm spacing) were placed in contact with the electrode surface, and the electrical resistance was measured using an RS Pro 804 digital ohmmeter (RS Components, UK; resolution 0.01 mΩ).

The electrical resistivity was calculated according to the Smits four-point probe method.[2]

$$\rho = (\pi / \ln 2) \cdot C \cdot t \cdot R$$

where (C) is the geometrical correction factor (0.9973), (t) is the thickness of the coated electrode, and (R) is the measured resistance. The effective electrical conductivity was then calculated as,

$$\sigma = 1/\rho$$

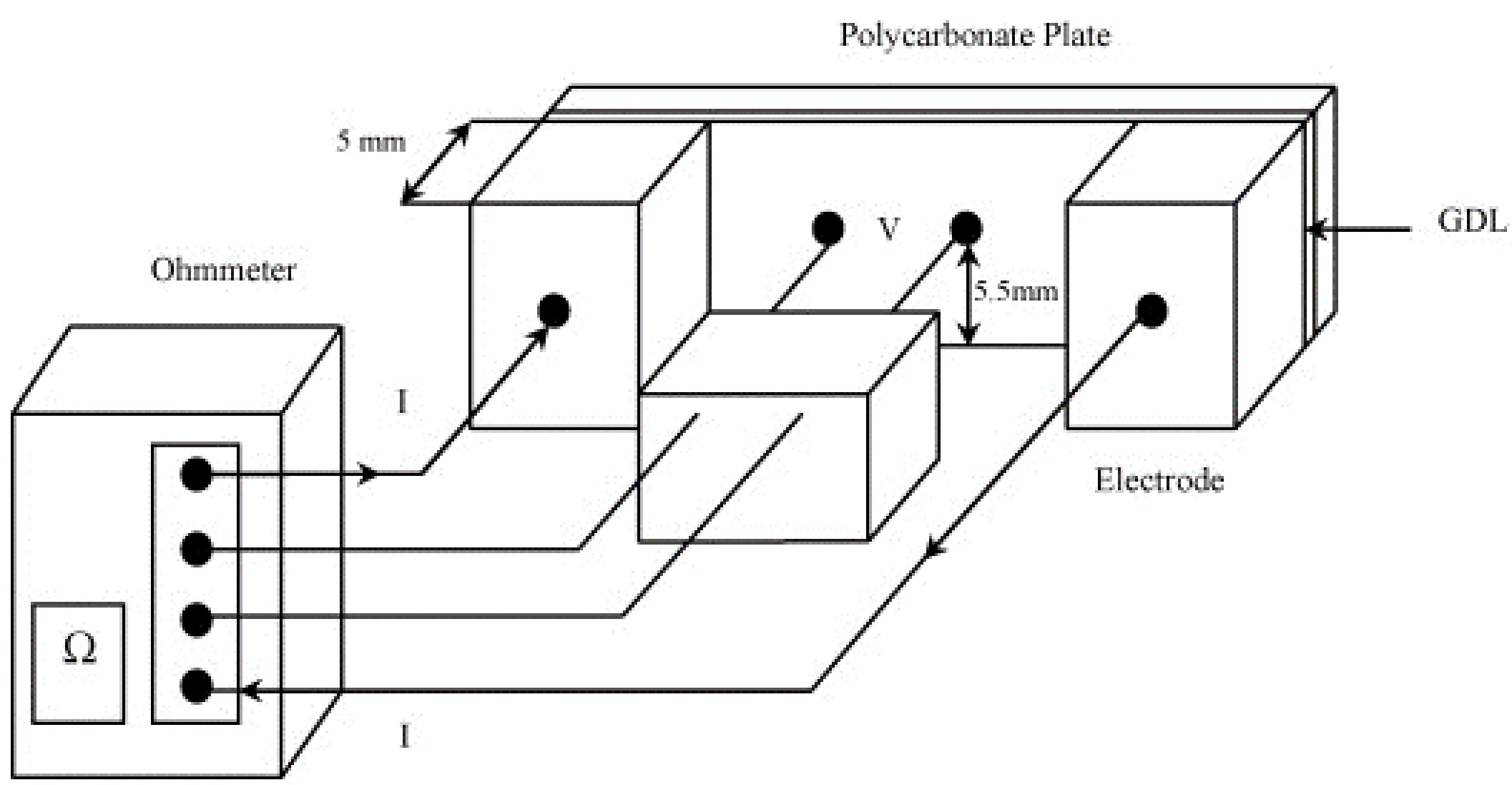

Figure S 7 A schematic diagram of the in-plane electrical conductivity test device.

For the phytic acid-doped electrode, the measured resistance was 0.394 Ω, corresponding to an effective in-plane electrical conductivity of 2.81×10^3 S m^{-1} (28.1 S cm^{-1}). For comparison, an undoped (pristine emeraldine base) PANi-coated electrode prepared and measured under identical conditions did not yield a stable, measurable resistance within the range of the instrument, consistent with the much lower conductivity

expected for the undoped emeraldine base form. This pronounced contrast provides further qualitative evidence that phytic acid doping substantially enhances charge transport in PANi.

(1) Ismail, M. S.; Damjanovic, T.; Ingham, D. B.; Pourkashanian, M.; Westwood, A. Effect of polytetrafluoroethylene-treatment and microporous layer-coating on the electrical conductivity of gas diffusion layers used in proton exchange membrane fuel cells. *Journal of Power Sources* **2010**, *195* (9), 2700-2708. DOI: https://doi.org/10.1016/j.jpowsour.2009.11.069.
(2) Smits, F. M. Measurement of Sheet Resistivities with the Four-Point Probe. *Bell System Technical Journal* **1958**, *37* (3), 711-718. DOI: https://doi.org/10.1002/j.1538-7305.1958.tb03883.x (acccessed 2026/06/29).
